# Vital Analysis of Cryopreserved Sperm of Marbled Flounder, *Pseudopleuronectes yokohamae*

**DOI:** 10.3389/fphys.2021.696737

**Published:** 2021-06-28

**Authors:** Shaharior Hossen, Soo Cheol Kim, Yusin Cho, Kang Hee Kho

**Affiliations:** Department of Fisheries Science, College of Fisheries and Ocean Sciences, Chonnam National University, Yeosu, South Korea

**Keywords:** cryopreservation, comet assay, motility, mitochondrial membrane potential, plasma membrane integrity, sperm

## Abstract

The marbled flounder (*Pseudopleuronectes yokohamae*) is a commercial flatfish in East Asia. The aim of this study was to improve its sperm cryopreservation protocol based on the vitality assessment of 7-day and 1-year cryopreserved sperm. Four extenders (extender-1: sucrose solution; extender-2: glucose solution; extender-3: fish Ringer's solution; and extender-4: modified fish Ringer's solution) were tested with a combination of five cryoprotectants (CPAs) (dimethyl sulfoxide: Me_2_SO; glycerol: GLY; ethylene glycol: EG; propylene glycol: PG; and methanol: MeOH) at four different concentrations (5, 10, 12, and 15%). Fluorescent technique was applied to detect the plasma membrane integrity (PMI), mitochondrial membrane potential (MMP), and DNA integrity of fresh and cryopreserved sperm specimens. Fresh sperm was diluted at a ratio of 1:2 (sperm:extender). Post-thaw motility of sperm cryopreserved using 15% Me_2_SO along with either extender-1 (86.0 ± 5.2%) or extender-2 (85.7 ± 7.1%) was similar (*p* > 0.05) to that of fresh sperm. Sperm cryopreserved using 12% GLY combined with extender-1 (83.67 ± 6.7%) or extender-2 (83.3 ± 4.7%) showed a similar motility to those cryopreserved with 15% Me_2_SO, but significantly lower from fresh sperm. The type of straw (0.25 or 0.50 mL) did not show any significant difference (*p* > 0.05) in post-thaw sperm motility. The highest values of PMI and MMP were observed for 7-day cryopreserved sperm using extender-1 in combination with 15% Me_2_SO (91.0 ± 2.9% and 90.0 ± 2.0%, respectively) or 12% GLY (90.0 ± 1.3% and 90.0 ± 4.6%, respectively). These results were similar to those of fresh sperm (95.3 ± 2.1% and 92.9 ± 2.5%, respectively). PMI and MMP of 1-year cryopreserved sperm using extender-1 in combination with 15% Me_2_SO (90.3 ± 2.5% and 89.3 ± 2.1%, respectively) or 12% GLY (90.0 ± 4.4% and 88.7 ± 2.2%, respectively) were significantly similar (*p* > 0.05) to those of fresh sperm. Sperm DNA integrity did not reveal any significant difference (*p* > 0.05) between fresh and cryopreserved (7-day and 1-year) sperm. Based on the assessed sperm vitality indicators, a cryopreservation protocol using extender-1 in combination with 15% Me_2_SO or 12% GLY has potential for hatchery as well as to create a germplasm bank.

## Introduction

The marbled flounder (*Pseudopleuronectes yokohamae*) is a commercially important species in Korea (Park et al., 2016), China (Liu et al., 2015), and Japan (Kusakabe et al., 2017). It is considered a valuable flatfish for coastal fisheries in Korea and Japan (Kusakabe et al., 2017). Although aquaculture technology for this species has been developed in Japan (Lucas et al., 2019), it has not been reported from other countries yet. Gonadal maturity occurs earlier in males than in females of this species (Kim et al., 2016), making its hatchery production difficult. Cryopreserved sperm can solve this problem by supplying sperm during hatchery production (Hassan et al., 2017; Gheller et al., 2019).

Sperm cryopreservation is an important method of storing genetic materials (Nahiduzzaman et al., 2011) and providing a constant supply of sperm (Kim et al., 2020a). This technique provides a stable source of sperm for artificial fertilization, selective breeding (Viveiros et al., 2012), and rapid sperm transfer among hatcheries (Xin et al., 2018a; Cejko et al., 2020). The cryopreservation protocol should optimize several factors, including sperm collection, sperm dilution (sperm:extenders), selection of cryoprotectants (CPAs), equilibration time, freezing in liquid nitrogen (LN), and thawing (Yang et al., 2017; Judycka et al., 2019). An extender can act as an artificial seminal plasma for sperm cryopreservation and reduce the metabolic activity of sperm (Sarosiek et al., 2016; Cejko et al., 2020). The extender is necessary to dilute sperm, and a CPA is required to protect sperm against damage during the freezing step (Suquet et al., 2000; Immerman and Goetz, 2014). The success of cryopreservation depends on both CPA type and concentration (Fernández-Santos et al., 2006; Soni et al., 2019). Recently, marine fish sperm cryopreservation has been accomplished using intracellular CPAs such as glycerol (GLY), dimethyl sulfoxide (Me_2_SO), methanol (MeOH), ethylene glycol (EG), and propylene glycol (PG). GLY (10–20%), Me_2_SO (10–20%), and PG (10%) are considered the most effective CPAs for the cryopreservation of teleost sperm (Cabrita et al., 2010). Currently, sperm cryopreservation techniques exist for over 200 fish species (Nahiduzzaman et al., 2011). Cryopreservation of sperm of several flounder species has been reported (Zhang et al., 2003; Lanes et al., 2008; Tian et al., 2008; Brown et al., 2012; Liu et al., 2015). However, very little is known about the cryopreservation of marbled flounder sperm. In addition, previous studies did not address the effects of the short-term and long-term cryopreservation on the post-thaw vitality of marbled flounder sperm (Song et al., 2016). Evaluation of the post-thaw vitality of the long-term cryopreserved sperm is needed for storage and commercial applications (Kim et al., 2020a).

Cryopreservation and thawing can decrease the integrity of the acrosome and plasma membrane. They can also reduce the motility and viability of sperm of all species (Xin et al., 2018a; Soni et al., 2019). Fluorescent technique is an important method of evaluating the sperm viability (He and Woods, 2004; Pereira et al., 2010). Recently, this technique has been used to assess the plasma membrane integrity (PMI), mitochondrial membrane potential (MMP), and acrosome integrity (AI) of sperm (Celeghini et al., 2007; Pereira et al., 2010; Hossen et al., 2021a,b). Post-thaw sperm motility (Agnihotri et al., 2016; Balamurugan et al., 2019) and MMP (Agnihotri et al., 2016) are the indicators of cryopreserved sperm quality. MMP indicates the mitochondrial energy status. It directly regulates the motility of sperm (Agnihotri et al., 2016). DNA integrity suggests the success of cryopreservation (Balamurugan et al., 2019). It can be detected using a comet assay (single-cell gel electrophoresis). The aim of this study was to improve the sperm cryopreservation technique and evaluate the post-thaw vitality of 7-day and 1-year cryopreserved marbled flounder sperm. The effects of (i) different concentrations of CPAs, (ii) different extenders, (iii) different types of straws, and (iv) different CPAs on PMI, MMP, and DNA integrity of cryopreserved sperm were observed to optimize the cryopreservation protocol of marbled flounder, *P. yokohamae*.

## Materials and Methods

Experimental protocols used were approved by Chonnam National University Animal Care and Use Committee (Approval No.: CNU IACUC-YS-2019-10; Approval date: December 10, 2019). All fish experiments were performed according to the Guidelines for the Care and Use of Laboratory Animals of the National Institutes of Health.

### Sperm Collection

Five sexually mature male marbled flounder were collected from Yeosu special fisheries market (Yeosu, Korea) in January and February of 2020 during the natural peak of its breeding season. Three to five individuals were previously used for the successful sperm cryopreservation of Atlantic halibut, *Hippoglossus hippoglossus* (Babiak et al., 2006); sterlet, *Acipenser ruthenus* (Xin et al., 2018b); striped catfish, *Pseudoplatystoma magdaleniatum* (Herrera-Cruz et al., 2019); and brown Trout (Rusco et al., 2020). Before the sperm collection, the area surrounding the genital pore was dried with a paper towel. Sperm was collected into 5-mL macro-tubes by gently stripping sperm ducts of fish. Special care was taken to prevent the contamination with blood, mucus, and urine. The abdomen of each fish was stripped to collect all possible sperm. These macro-tubes were immediately placed on ice after the sperm collection.

### Quality Evaluation of Fresh Sperm

Sperm quality was assessed based on the sperm motility, PMI, and MMP to ensure the quality of fresh sperm. Sperm motility was observed according to the method described by Ding et al. (2009), Ahn et al. (2018), and Muchlisin et al. (2020). Briefly, an aliquot of 10 μL sperm was diluted with 100 μL of filtered seawater in a microtube. Subsequently, 2 μL of the diluted sperm was added to 100 μL of filtered seawater on a glass slide. Immediately, sperm were video-recorded using a microscope (Nikon Eclipse E600) with a 40x objective lens. Motility was calculated three times using three different samples (*n* = 3). Sperm survival rate was immediately assessed using a LIVE/DED® sperm viability kit. MMP was examined using the Rh123/PI® method. The density of fresh sperm was calculated using a hemocytometer (Laboratory Glassware, Germany).

### Apparatus

Styrofoam boxes (25.0 ×25.0 ×21.0 cm) with a rack height of 6 cm were used to conduct the cryopreservation experiments. LN with a height of 5 cm was placed into Styrofoam boxes. The rack height was maintained from the surface of the LN. A digital water bath (JISICO Lab and Scientific Instruments) was used to thaw cryopreserved straws.

### Cryopreservation and Storage of Marbled Flounder Sperm

**Figure 1:**
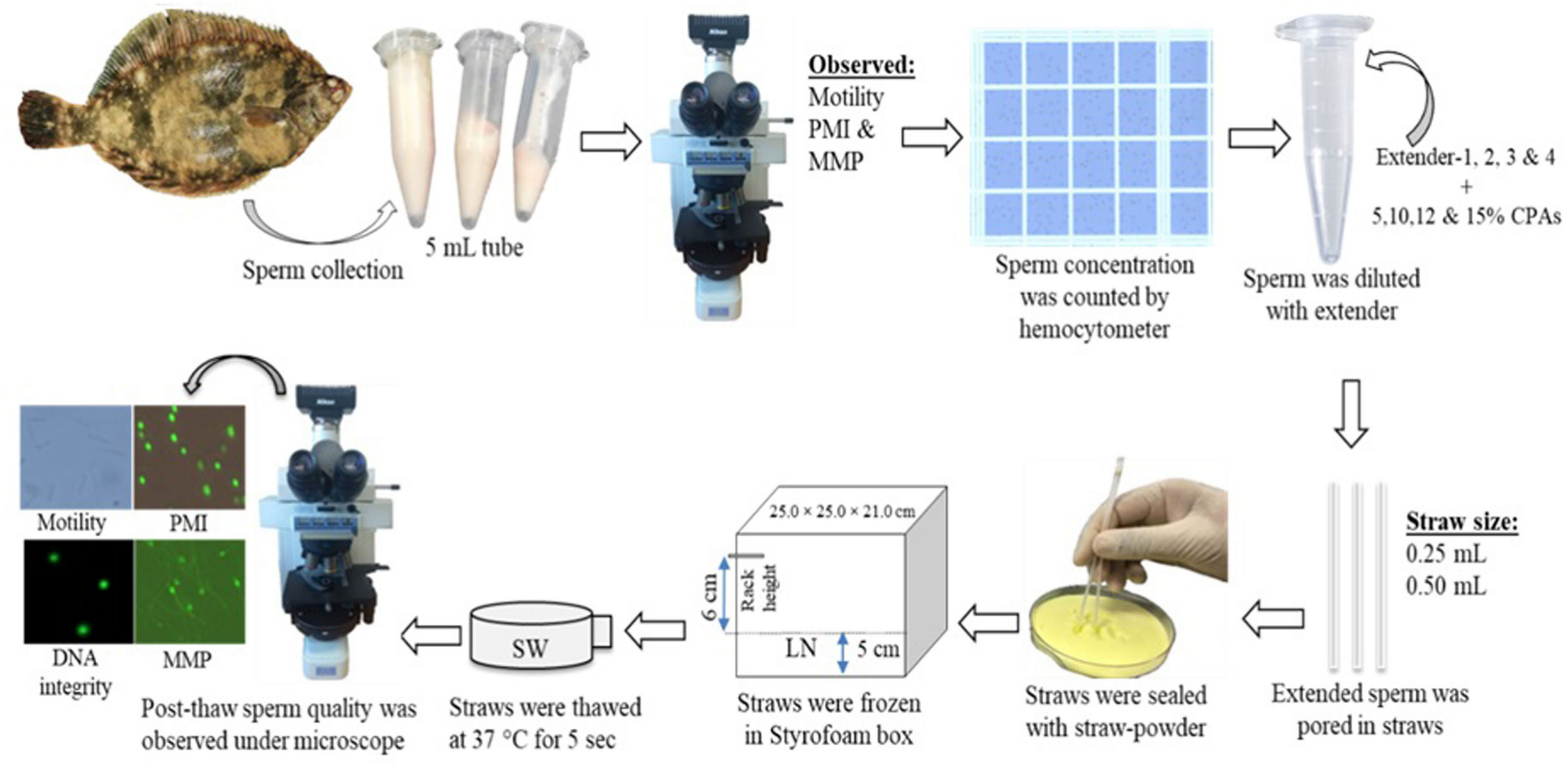
Flow diagram showing the cryopreservation technique used for marbled flounder sperm.

The following basic cryopreservation protocol was used in the present study (Figure 1). Sperm were diluted with extender solution and CPAs. The working solution (sperm with extender solution) was mixed gently in microtubes and placed into straws (0.25 and 0.50 mL). Straws (sealed with straw powder) were placed into Styrofoam boxes for the initial freezing. These boxes were covered for 10 min. Subsequently, straws were immediately submerged into LN for at least 3 h. For 1-year preservation, straws were stored in a 38-L LN tank (model: 38VHC-11M, serial: 80907, Worthington Industries, United States). It was observed that the height of LN in the tank decreased 5.5 cm in every week, and the tank was refilled with LN. Straws were thawed at 37°C for 5 s in seawater (SW) within 4 s. Thawed sperm were transferred to microtubes and observed under a microscope to assess the sperm motility. Sperm samples were activated with the filtered SW to assess the sperm motility as described in section Quality Evaluation of Fresh Sperm.

### Experiment 1: Effects of Extenders and Cryoprotectants

Four extenders were evaluated in this study: (1) extender-1 (sucrose solution), 110 mM sucrose, 100 mM KHCO_3_, and 10 mM HEPES, pH 8.2; (2) extender-2 (glucose solution), 83.25 mM glucose, 136.89 mM NaCl, and 8.72 mM KCl, pH 7.9; (3) extender-3 (fish Ringer's solution), 154 mM NaCl, 5.64 mM KCl, 2.2 mM CaCl_2_·6H_2_O, and 3.8 mM NaHCO_3_; (4) extender-4 (modified fish Ringer's solution), 65 mM sucrose, 154 mM NaCl, 5.64 mM KCl, 2.2 mM CaCl_2_·6H_2_O, and 3.8 mM NaHCO_3_. The following five CPAs were used to evaluate their effects on the post-thaw sperm motility: Me_2_SO, EG, PG, GLY, and MeOH. These CPAs were used at the final concentrations of 5, 10, 12, and 15% with each extender based on the published results for other flounder species (Zhang et al., 2003; Lanes et al., 2008; Ding et al., 2011; Brown et al., 2012; Hu et al., 2016). Working solutions were prepared by mixing different CPA concentrations with extenders and chilled at 4°C for ~24 h prior to use.

### Experiment 2: Effects of Dilution Ratios

Two dilution ratios (1:2 and 1:4) were used to establish the optimal cryopreservation technique based on the published results for other flounder species (Zhang et al., 2003; Tian et al., 2008; Brown et al., 2012). Two concentrations (10 and 12%) of CPAs (Me_2_SO and GLY) with extender-1 and extender-2 were used to select the best dilution ratio for the effective cryopreservation.

### Experiment 3: Effects of Straw Size

Effects of straw size were evaluated by comparing the post-thaw sperm motility of sperm cryopreserved with 0.25 or 0.50 mL straws. This experiment was designed using the best concentration of CPAs and extenders selected from Experiment 1 and the best dilution ratio from Experiment 2.

### Experiment 4: Fluorescent Technique Used to Assess Fresh and Cryopreserved Sperm Vitality

#### Plasma Membrane Integrity

Plasma membrane integrity values of fresh and cryopreserved sperm (7 days and 1 year) were assessed using a LIVE/DEAD® sperm viability kit (Invitrogen Molecular Probes, Eugene, OR, United States), which contained two fluorescent dyes, SYBR-14® and propidium iodide (PI), to facilitate the accurate and rapid assessment of PMI (Horváth et al., 2008; Merino et al., 2012; Hossen et al., 2021a,b). Briefly, 10 μL of post-thaw sperm was mixed with 990 μL of 1X PBS (phosphate-buffered saline) and tapped slightly to ensure proper dilution. An aliquot of 5 μL SYBR-14® was mixed with a sample and incubated at 37°C for 10 min in the dark followed by mixing with 10 μL PI. After slight tapping and incubating at 37°C in the dark again for 10 min, a 2 μL aliquot of the sample was then placed on a glass slide and covered with a cover slip. Samples were immediately observed under a fluorescent microscope (Nikon Eclipse E600). A green filter (excitation filter 450–490 nm) was used to capture images of green-stained live sperm (SYBR-14^+^/PI^**−**^), and a red filter (emission filter 510–560 nm) was used to capture images of red-stained dead sperm (SYBR-14^**−**^/PI^+^). Images captured with these green and red filters were merged with images taken without a filter to assess the sperm viability and PMI. Three replications (*n* = 3) were considered to analyze the PMI values, and a minimum of 200 sperm cells were considered in each replication.

#### Mitochondrial Membrane Potential

Mitochondrial membrane potentials of cryopreserved sperm (7 days and 1 year) were evaluated using Rh123/PI® (Sigma-Aldrich Pty Ltd.) using a protocol described in the study by Kim et al. (2020b) with slight modifications. Briefly, 25 μL of post-thaw sperm was mixed with 975 μL of 1 × PBS and slightly tapped for appropriate dilution. Then 1 μL aliquot of Rh123 was mixed with the sample and incubated at 20°C in the dark for 10 min. Next, 5 μL PI was added followed by the slight tapping. The sample was incubated at 20°C in the dark again for 10 min. Then 2 μL aliquot of the sample was placed on a glass slide and covered with a cover slip. Samples were immediately observed under a fluorescent microscope (Nikon Eclipse E600). A green filter (excitation filter 450–490 nm) was used to capture images of green-stained intact mitochondrial membranes (Rh123^+^/PI^**−**^), and a red filter (emission filter 510–560 nm) was used to capture images of red-stained damaged mitochondrial membranes (Rh123^**−**^/PI^+^). Images captured with these green and red filters were merged with images taken without a filter to assess MMPs of cryopreserved sperm. Three replications (*n* = 3) were considered to analyze the PMI values, and a minimum of 200 sperm cells were considered in each replication.

### Experiment 5: Comet Assay to Detect Integrity of Sperm DNA

Comet assay (single-cell gel electrophoresis) of fresh and cryopreserved sperm (7 days and 1 year) was performed using the protocol described in the study by Kim et al. (2020a) with slight modifications using a Comet Assay® kit (Trevigen Inc., Gaithersburg, MD, United States). Briefly, sperm were suspended (1 ×10^5^ cells/mL) in pre-chilled 1 × PBS and immobilized in low-melting agarose gel on the comet slide. Slides were immersed in a lysis solution for 1 h and an alkaline unwinding solution for another 1 h at 4°C in the dark. These comet slides were electrophoresed at 21V for 30 min using a pre-chilled alkaline electrophoresis solution. Samples were stained with Vista green dye and then incubated in a dark chamber for 30 min. Slides were immediately visualized. Images were captured using a fluorescence microscope (excitation filter at 450–490 nm; Nikon Eclipse E600). A minimum of 100 cells were used to analyze the results of the comet assay. Comet Assay IV image analysis software (version 4.3.2, Perceptive Instruments Ltd., United Kingdom) was used to analyze the head length, tail length, percentage of DNA in the tail, tail moment, olive tail moment (OTM), and extent of tail moment.

### Statistical Analysis

All statistical analyses were performed using SPSS 16.00 software (SPSS Inc., Chicago, IL, United States). Statistical significance of results was determined using a non-parametric one-way ANOVA followed by Duncan's multiple range test. Differences were considered significant at *p* < 0.05. A GraphPad Prism software (GraphPad Prism version 5.00 for Windows, CA, United States) was used to generate graphs.

## Results

### Sperm Density

Male fish used for this study had an average (mean ±SD) body weight of 351.7 ± 53.5 g, a total length of 31.0 ± 1.4 cm, and a standard length of 26.1 ± 1.4 cm. Total milt volume was 8.3 ± 0.3 mL with a density of 18.0 ± 2.3 (×10^9^) cells/mL. The total number of counted cells was 15.0 ± 1.6 ( ×10^10^).

### Experiment 1: Effects of Extenders and Cryoprotectants

**Figure 2:**
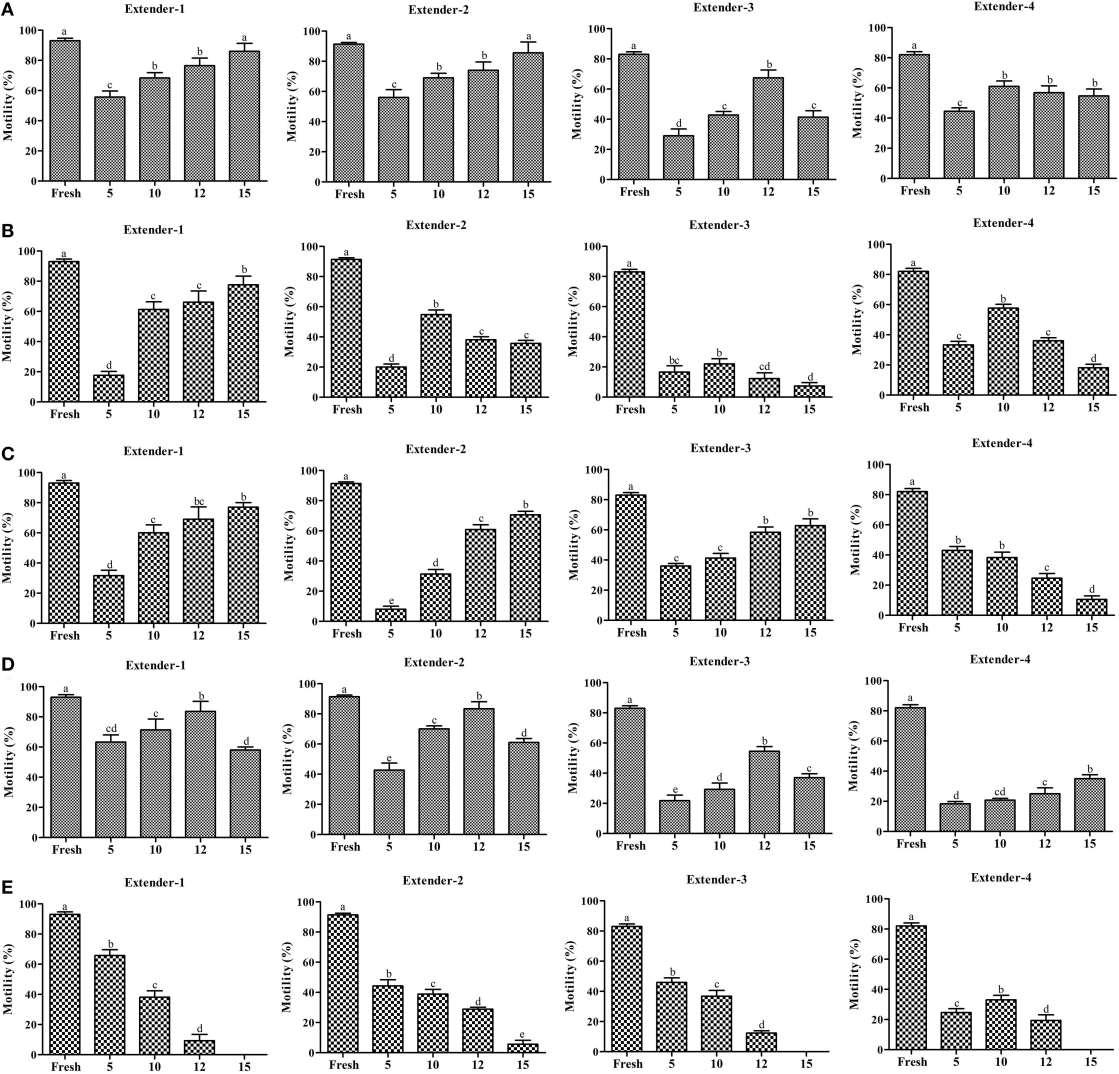
Post-thaw motility (*n* = 3) of sperm cryopreserved using different concentrations (5, 10, 12, and 15%) of cryoprotectants (Me_2_SO, EG, PG, GLY, and MeOH) combined with extenders (extenders-1,−2,−3, and −4). **(A)** Post-thaw motility of sperm cryopreserved using different concentration of Me_2_SO combined with different extenders; **(B)** post-thaw motility of sperm cryopreserved using different concentration of EG combined with different extenders; **(C)** post-thaw motility of sperm cryopreserved using different concentration of PG combined with different extenders; **(D)** post-thaw motility of sperm cryopreserved using different concentration of GLY combined with different extenders; **(E)** post-thaw motility of sperm cryopreserved using different concentration of MeOH combined with different extenders. Significant different levels (*p* < 0.05) are denoted by different letters.

Effects of extenders and CPAs on the post-thaw sperm motility are presented in Figure 2, Supplementary Table 1. The predominant post-thaw sperm motility was recorded using 15% Me_2_SO with extender-1 (86.0 ± 5.2%) or extender-2 (85.7 ± 7.1%). Post-thaw motility of sperm cryopreserved using extender-1 or extender-2 in combination with 15% Me_2_SO was similar to that of fresh sperm (*p* > 0.05) (Figure 2A).

Sperm cryopreserved using extender-1 combined with 15% EG (77.7 ± 5.7%) exhibited significantly higher (*p* < 0.05) post-thaw motility than other types of extender (Figure 2B). Sperm cryopreserved using EG exhibited lower post-thaw motility than those cryopreserved with Me_2_SO or GLY (Supplementary Table 1).

Post-thaw motility of sperm cryopreserved using extender-1 combined with 15% PG (77.0 ± 3.0%) exhibited significantly higher (*p* < 0.05) post-thaw motility than other types of extender in different concentrations of PG (Figure 2C).

Post-thaw motility of sperm cryopreserved using extender-1 (83.67 ± 6.7%) or extender-2 (83.3 ± 4.7%) in combination with 12% GLY was similar (*p* > 0.05) to that of sperm cryopreserved with 15% Me_2_SO, but lower from that of fresh sperm (Figure 2D; Supplementary Table 1).

Sperm cryopreserved using extender-1 combined with 5% MeOH (65.8 ± 3.9%) exhibited significantly higher (*p* < 0.05) post-thaw motility than other types of extender in different concentration of MeOH (Figure 2E). On the other hand, post-thaw motility was not observed when sperm were cryopreserved with 15% MeOH in combination with extender-1,−3, or−4 (Figure 2E).

### Experiment 2: Effects of Dilution Ratio

**Figure 3:**
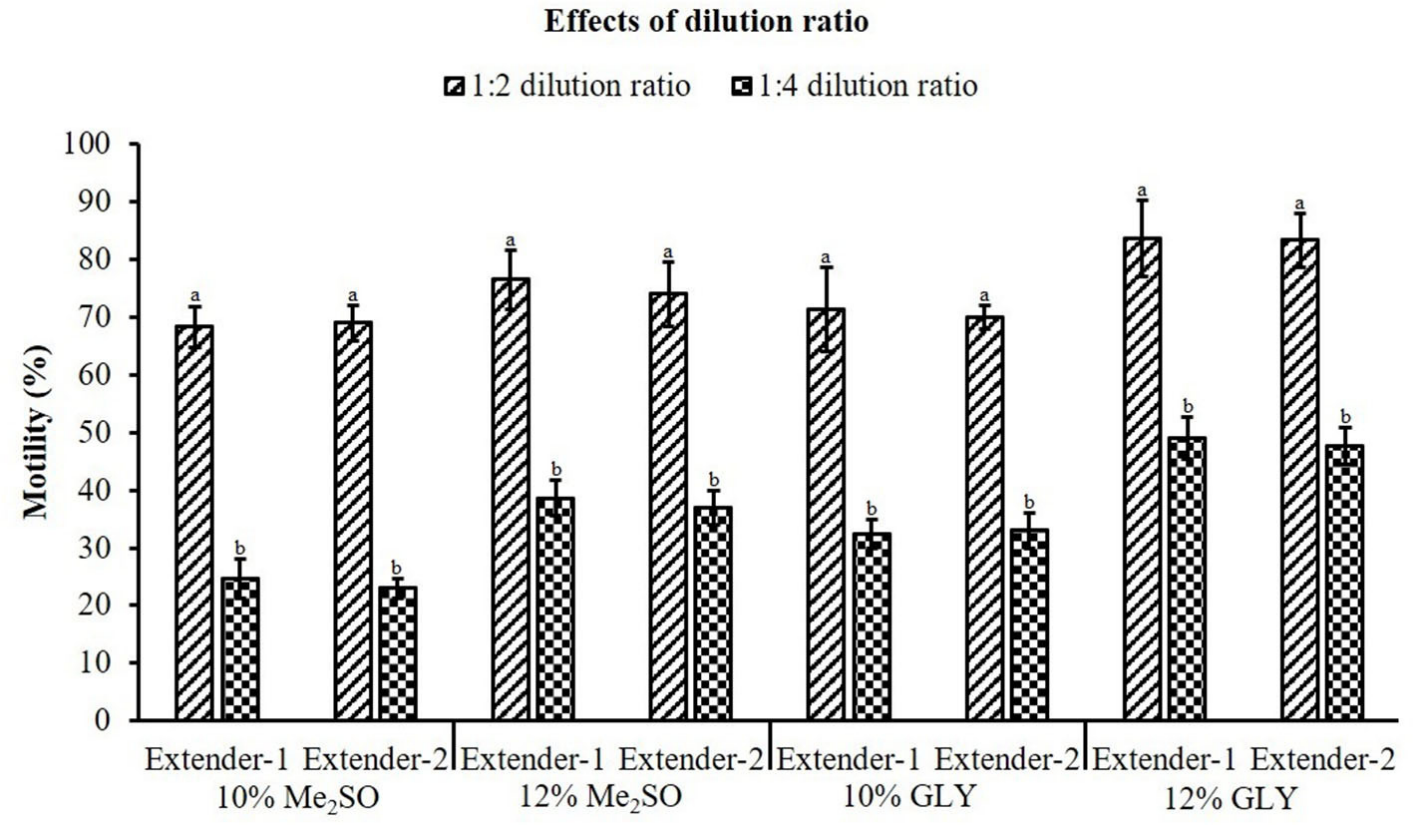
Effects of dilution ratio on motility of cryopreserved marbled flounder sperm (*n* = 3). Significant different levels (*p* < 0.05) are denoted by different letters.

In this experiment, all CPAs in combination with extender-1 or extender-2 showed significant differences between dilution ratios of 1:2 and 1:4 (Figure 3). Sperm diluted with extenders at a ratio of 1:2 showed significantly (*p* < 0.05) higher motility than those with extenders at a ratio of 1:4.

### Experiment 3: Effects of Straw Size

**Figure 4:**
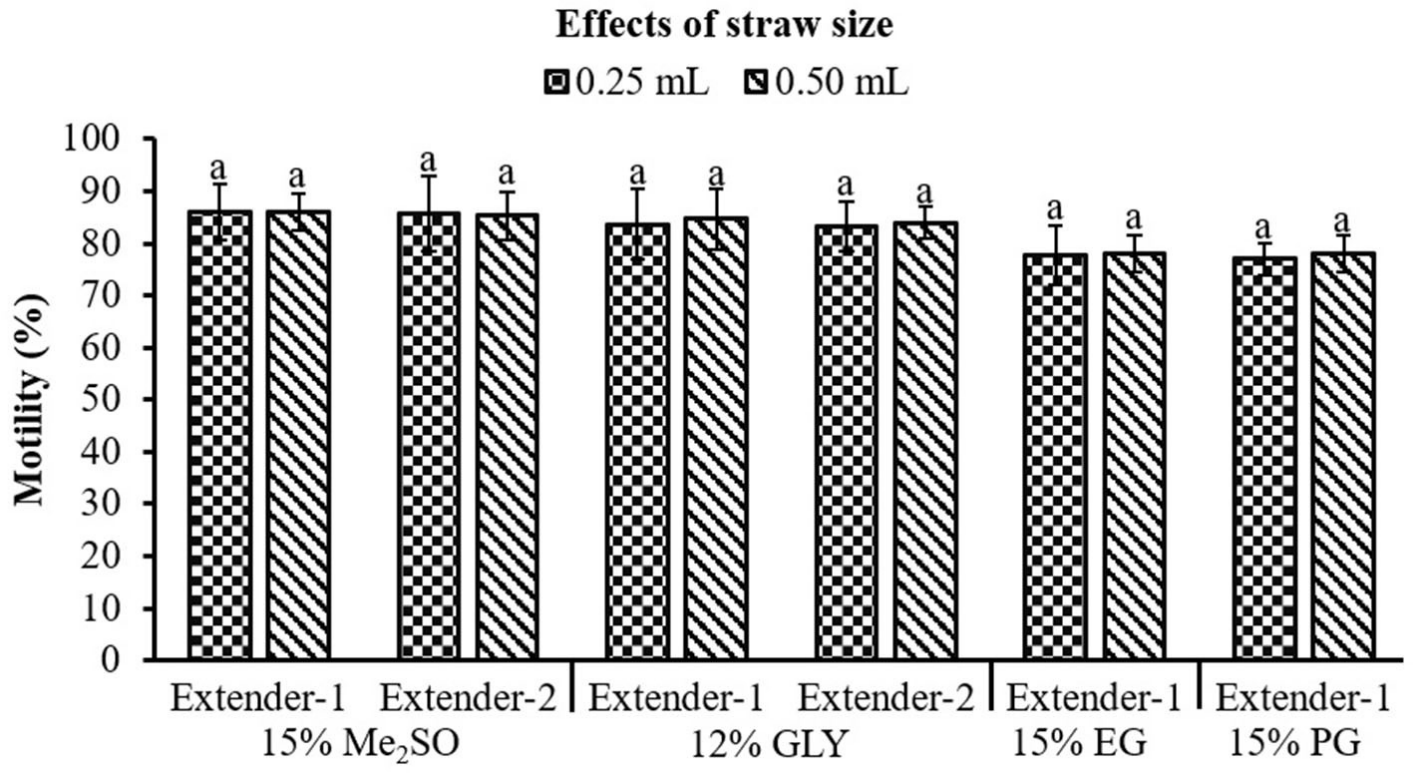
Effects of straw size (0.25 mL and 0.50 mL) on post-thaw motility of marbled flounder sperm (*n* = 3). Significant different levels (*p* < 0.05) are denoted by different letters.

Optimal combination of CPA and extender was selected based on the post-thaw motility results from Experiments 1 and 2. Sperm packaged in 0.25 mL straws and those packaged in 0.50 mL straws did not show a significant (*p* > 0.05) difference in post-thaw motility (Figure 4).

### Experiment 4: Fluorescent Technique for the Assessment of Cryopreserved Sperm Vitality

#### Plasma Membrane Integrity

**Figure 5:**
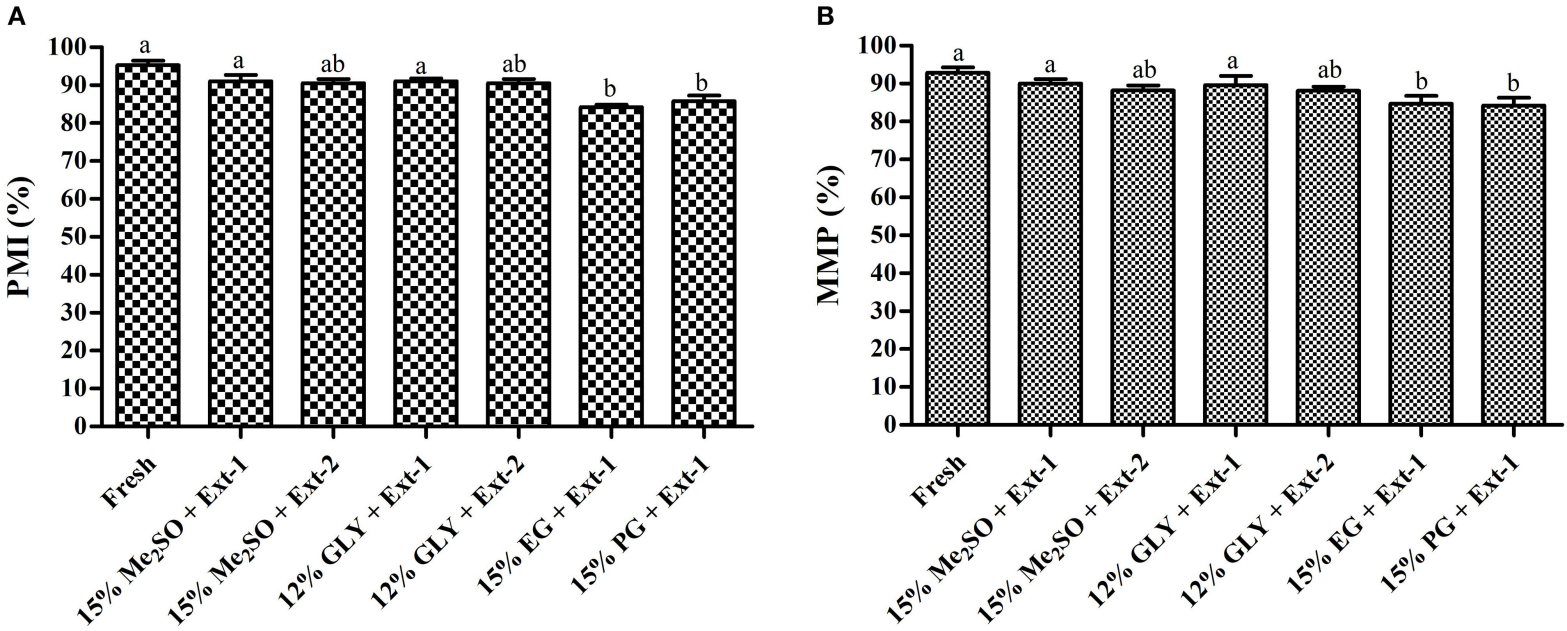
Plasma membrane integrity (%) and mitochondrial membrane potentials (%) of post-thaw marbled flounder sperm (*n* = 3). Significant different levels (*p* < 0.05) are denoted by different letters. **(A)** Plasma membrane integrity of cryopreserved sperm, **(B)** Mitochondrial membrane potentials of cryopreserved sperm.

**Figure 6:**
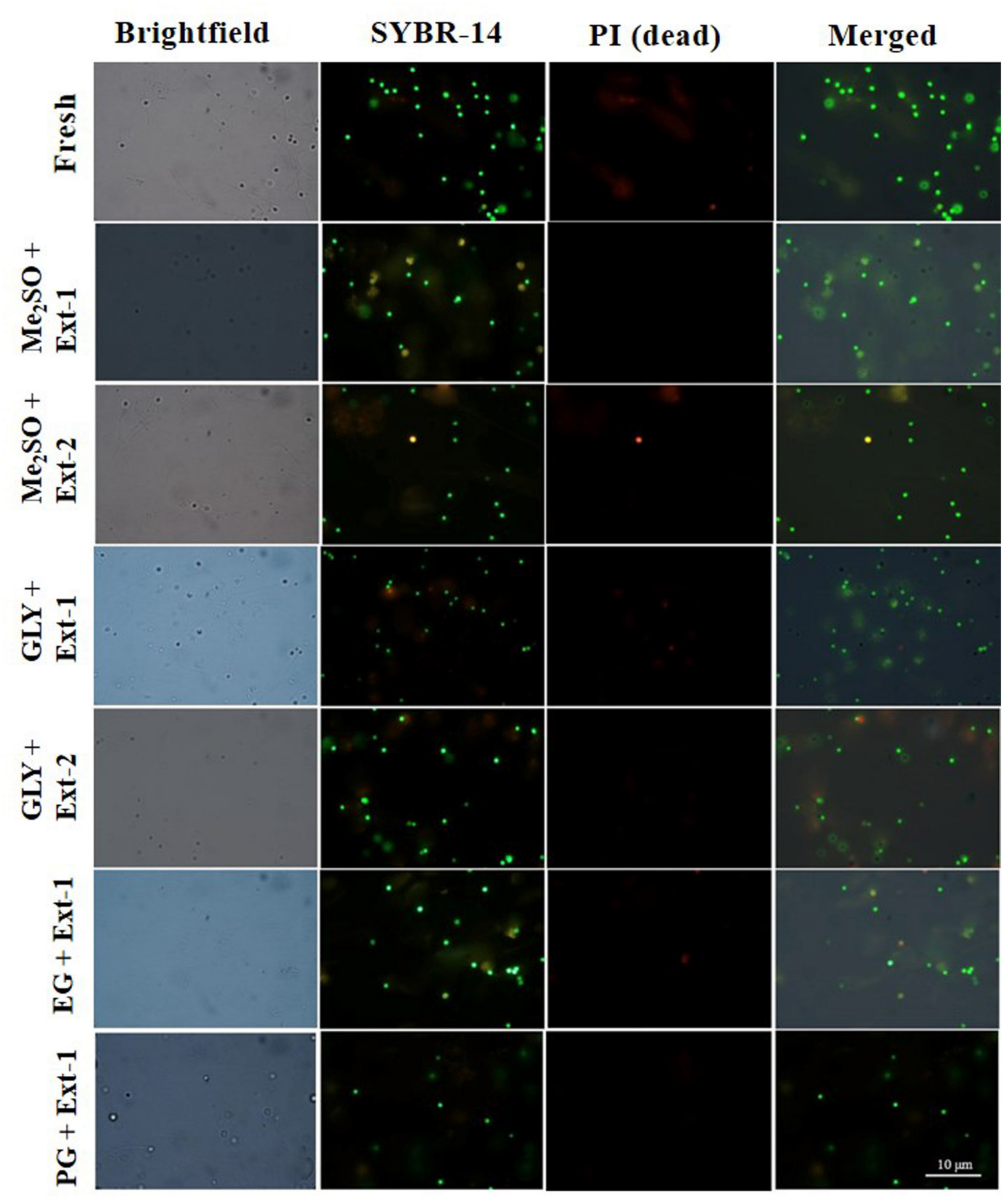
Fluorescent-stained photographs for detecting the plasma membrane integrity of cryopreserved marbled flounder sperm ( ×100 magnification).

Six combinations of CPAs and extenders were used to assess the PMI values of 7-day cryopreserved sperm based on the results of Experiment 1. PMI values (Figure 5A) of sperm cryopreserved with extender-1 in combination with 15% Me_2_SO (91.0 ± 2.9%), those of sperm cryopreserved with extender-1 in combination with 12% GLY (90 ± 1.3%), and those of fresh sperm (95.3 ± 2.1%) were not significantly different (*p* > 0.05). The morphology of cryopreserved sperm was similar to that of fresh sperm (Figure 6).

#### Mitochondrial Membrane Potential

**Figure 7:**
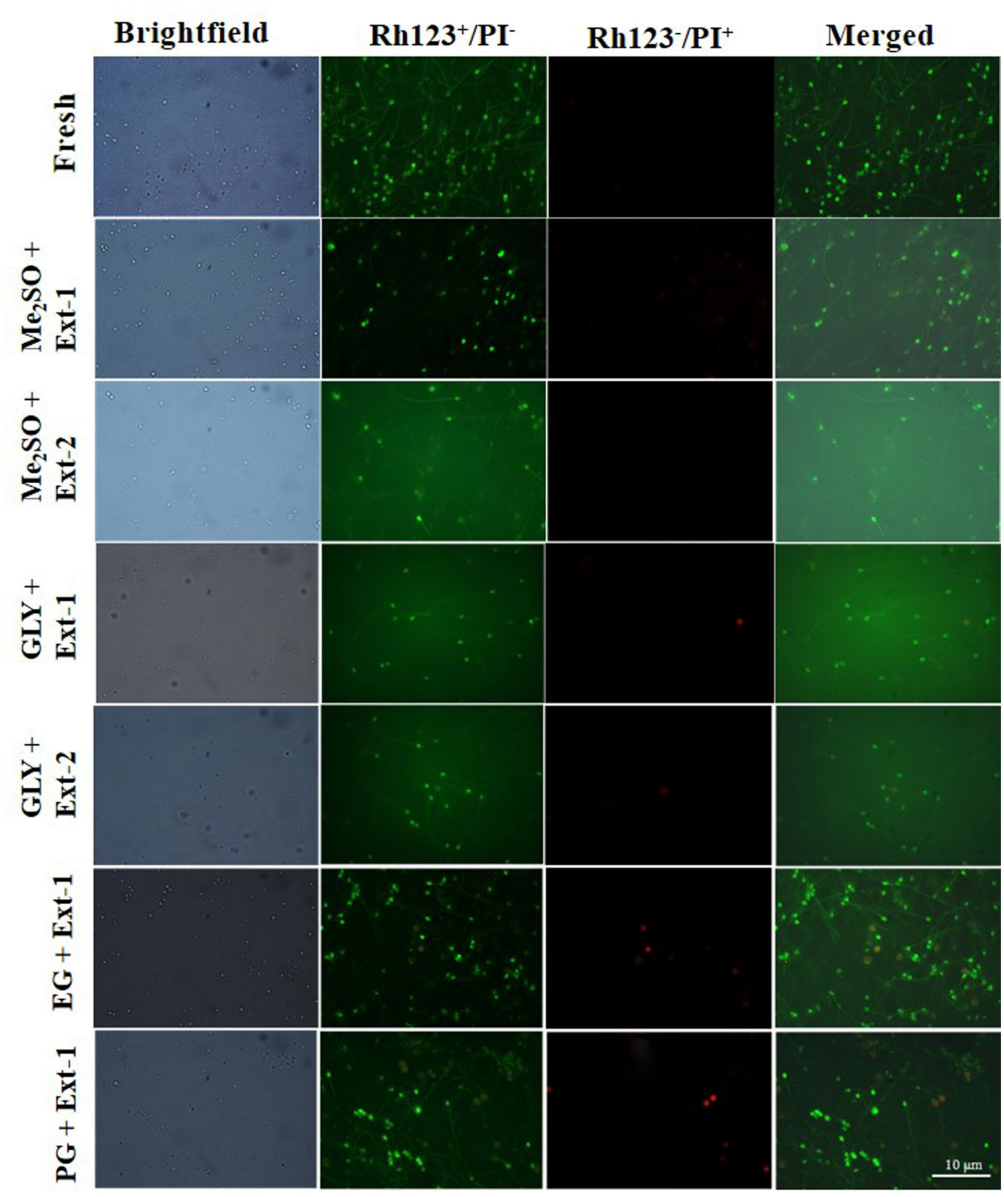
Fluorescent-stained photographs for detecting mitochondrial membrane potential of cryopreserved marbled flounder sperm ( ×100 magnification).

Based on the post-thaw motility index, six combinations of CPAs and extenders were used to assess MMPs of 7-day cryopreserved sperm. MMPs (Figure 5B) of sperm cryopreserved using extender-1 in combination with 15% Me_2_SO (90.0 ± 2.0%), those of sperm cryopreserved with extender-1 in combination with 12% GLY (90.0 ± 4.6%), and those of fresh sperm (92.9 ± 2.5%) did not show a significant difference (*p* > 0.05). The mitochondrial membrane of cryopreserved sperm was similar to that of fresh sperm (Figure 7).

### Experiment 5: Effects of 1-Year Cryopreservation on Post-thaw Sperm Vitality

**Figure 8:**
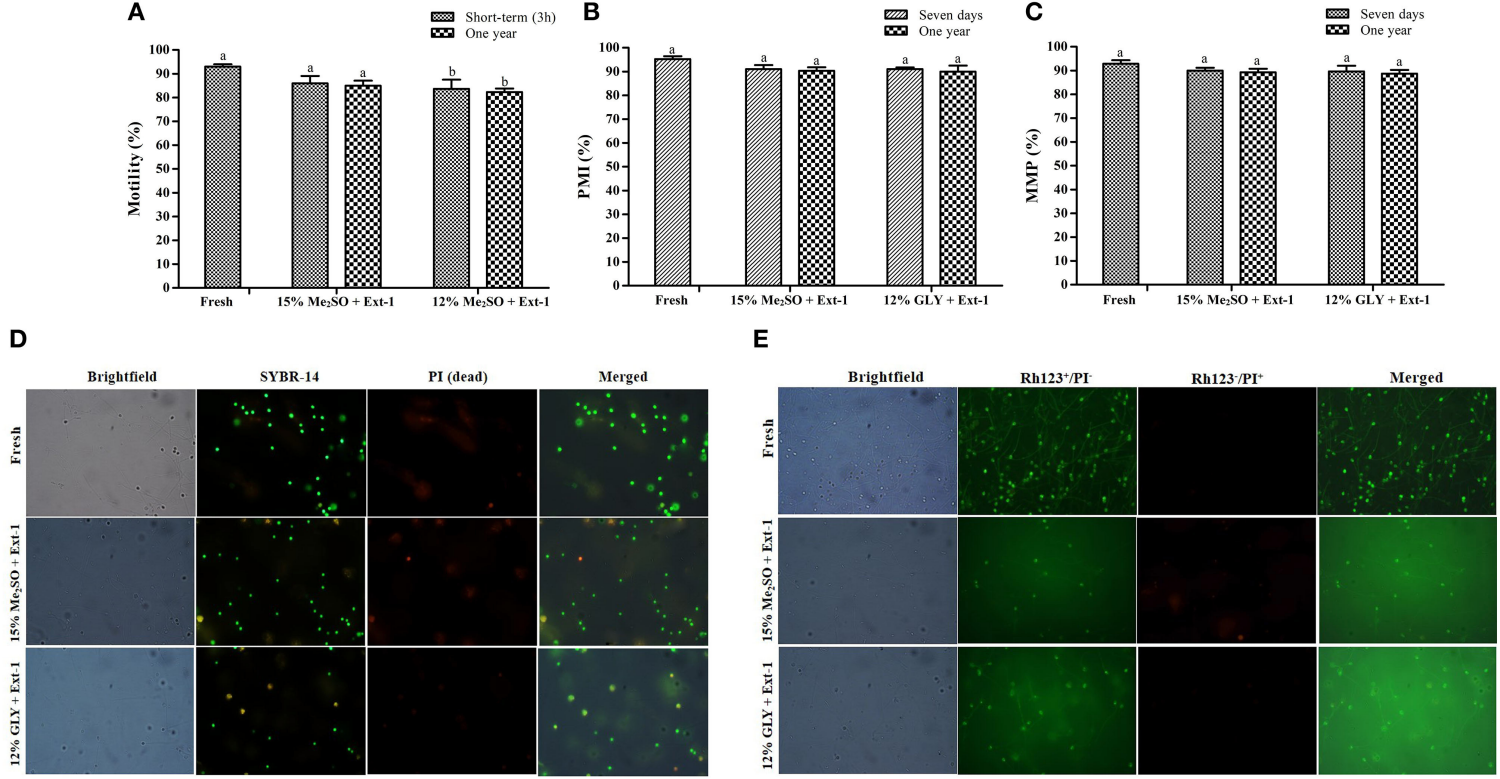
Effects of 1-year cryopreservation on post-thaw sperm vitality of marbled flounder. **(A)** Post-thaw sperm motility of 1-year cryopreserved sperm (*n* = 3); **(B)** plasma membrane integrity of 1-year cryopreserved sperm (*n* = 3); **(C)** mitochondrial membrane potential of 1-year cryopreserved sperm (*n* = 3); **(D)** fluorescent-stained photographs for detecting the plasma membrane integrity of 1-year cryopreserved marbled flounder sperm ( ×100 magnification). **(E)** Fluorescent-stained photographs for detecting the mitochondrial membrane potential of 1-year cryopreserved marbled flounder sperm ( ×100 magnification). Significant different levels (*p* < 0.05) are denoted by different letters.

Two combinations of CPAs and extenders (15% Me_2_SO + extender-1 and 12% GLY + extender-1) were used to evaluate the post-thaw vitality of 1-year cryopreserved sperm based on the results of Experiments 1 and 4. Post-thaw motility of 1-year cryopreserved sperm using 15% Me_2_SO + extender-1 (85.0 ± 3.6%) or 12% GLY + extender-1 (82.3± 2.5%) was significantly similar (*p* > 0.05) to that of immediate (3 h) cryopreserved sperm motility (Figure 8A). PMI values of 1-year cryopreserved sperm with extender-1 in combination with 15% Me_2_SO (90.3 ± 2.5%) or 12% GLY (90.0 ± 4.4%) did not show differences (*p* > 0.05) with those cryopreserved 7 days and those of fresh sperm (Figures 8B,D). MMP values of 1-year cryopreserved sperm with extender-1 in combination with 15% Me_2_SO (89.3 ± 2.1%) or 12% GLY (88.7 ± 2.2%) did not show differences (*p* > 0.05) with those cryopreserved 7 days and those of fresh sperm (Figures 8C,E).

### Experiment 6: Comet Assay to Determine Sperm DNA Integrity

**Figure 9:**
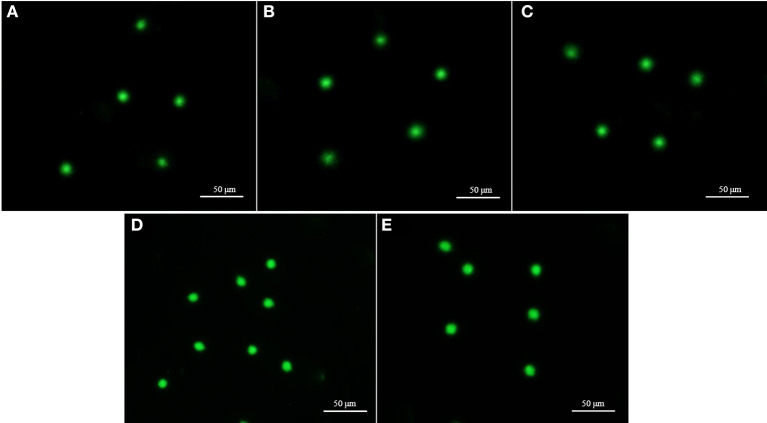
Fluorescent images of fresh and cryopreserved sperm after comet assay. Each comet represents the undamaged DNA in sperm. **(A)** Intact nuclei of fresh sperm. **(B)** Intact nuclei of 7-day cryopreserved sperm using extender-1 in combination with 12% GLY. **(C)** Intact nuclei of 7-day cryopreserved sperm using extender-1 in combination with 15% Me_2_SO. **(D)** Intact nuclei of 1-year cryopreserved sperm using extender-1 in combination with 12% GLY. **(E)** Intact nuclei of 1-year cryopreserved sperm using extender-1 in combination with 15% Me_2_SO. The comet gel was stained with Vista green dye.

Parameters of the comet assay for fresh and cryopreserved (7 days and 1 year) sperm are listed in Table 1. The percentage of DNA in the tail of 7-day cryopreserved sperm using extender-1 in combination with 12% GLY or 15% Me_2_SO was 0.7 ± 0.1% or 0.9 ± 0.1%, respectively. The percentage of DNA in the tail of 1-year cryopreserved sperm using extender-1 in combination with 12% GLY (0.9 ± 1.2%) or 15% Me_2_SO (0.9 ± 1.2%) did not show significant differences with those 7-day cryopreserved or fresh sperm. Intact DNA was seen for cryopreserved sperm and fresh sperm (Table 1). Comet assay results did not reveal any significant (*p* > 0.05) difference in DNA integrity between fresh and cryopreserved (7-day and 1-year) marbled flounder sperm (Figure 9).

**Table 1:** Results of comet assay parameters (mean ± standard deviation) of fresh and cryopreserved sperm (7-day and 1-year) samples.

**Parameters**	**Fresh sperm**	**Seven days**		**One year**	
		**Extender-1 + 12% GLY**	**Extender-1 + 15% Me_**2**_SO**	**Extender-1 + 12% GLY**	**Extender-1 + 15% Me_**2**_SO**
Head length (μ)	32.3 ± 4.3^a^	33.3 ± 4.6^a^	34.9 ± 2.8^a^	33.4 ± 4.4^a^	35.0 ± 3.6^a^
% DNA in head	99.4 ± 2.6^a^	99.3 ± 1.2^a^	99.1 ± 1.5^a^	99.1 ± 1.2^a^	99.1 ± 1.2^a^
Tail length (μ)	13.6 ± 4.8^a^	13.6 ± 2.5^a^	14.7 ± 2.1^a^	14.2 ± 3.8^a^	15.4 ± 5.2^a^
% DNA in tail	0.6 ± 0.1^a^	0.7 ± 0.1^a^	0.9 ± 0.1^a^	0.9 ± 1.2^a^	0.9 ± 1.2^a^
Tail moment (μ)	0.1 ± 0.0^a^	0.1 ± 0.0^a^	0.1 ± 0.0^a^	0.1 ± 0.0^a^	0.1 ± 0.0^a^
Olive tail moment (μ)	1.4 ± 0.2^a^	1.4 ± 0.3^a^	1.5 ± 0.3^a^	1.4 ± 0.4^a^	1.5 ± 0.4^a^
Extent tail moment (μ)	8.2 ± 1.2^a^	9.5 ± 1.4^a^	13.2 ± 1.2^b^	12.8 ± 1.3^a^	13.8 ± 1.3^b^

*Values are expressed as mean ± SD. Same letters (a, b) indicate no significant difference in values (p > 0.05)*.

## Discussion

The development of an effective sperm cryopreservation technique is a vital goal because of its significance for the conservation of biodiversity, reduced inbreeding, and minimization of domestic selection. Cryopreservation of fish sperm is affected by factors such as extenders, CPAs, dilution ratio, freezing process, and thawing process (Ahn et al., 2018). Present study improved a cryopreservation technique for marbled flounder sperm by optimizing these factors. In the present study, a sucrose base solution was used as extender-1. It had a composition similar to the extender used for the cryopreservation of sperm of summer flounder, *Paralichthys dentatus* (Brown et al., 2012); Brazilian flounder, *Paralichthys orbignyanus* (Lanes et al., 2008); and spotted halibut, *Verasper variegatus* (Tian et al., 2008). Glucose solution was used as extender-2. It was similar to the solution used for the cryopreservation of sperm of *P. yokohamae* (Song et al., 2016). Ringer's solution (extender-3) and modified Ringer's solution (extender-4) were also used in this study to compare their effects on the post-thaw sperm motility. The optimal dilution ratio of extender to sperm is species-specific (Ding et al., 2011). The present study showed a significant difference in the post-thaw sperm motility between two dilution ratios (1:2 and 1:4), similar to the results reported for sperm of *H. hippoglossus* (Ding et al., 2011) and *Paralichthys dentatus* (Brown et al., 2012).

In the present study, five common types of CPAs at four different concentrations were used for the cryopreservation of marbled flounder sperm. These CPAs used in the present study are categorized as penetrating CPAs (Cabrita et al., 2010; Iorio et al., 2019). All of them act with a similar principle (Iorio et al., 2019). CPAs can prevent ice crystallization during the freeze–thaw process (Fuller, 2004; Yang et al., 2010).

The present study showed the significant effects of extenders and CPAs on the motility of post-thaw sperm. In the present study, 15% Me_2_SO and 12% GLY had similar (*p* > 0.05) effects on the post-thaw sperm motility when they were combined with extender-1 and extender-2, respectively. Cryopreservation using Me_2_SO or GLY also resulted in the highest post-thaw motility of sperm of *Paralichthys olivaceus* (Zhang et al., 2003) and *Paralichthys dentatus* (Brown et al., 2012; Liu et al., 2015). This is likely because sugars such as sucrose and glucose can stabilize phospholipids in the cell membrane during the freezing step (Ahn et al., 2018). Sugars may supply energy, reduce ice crystallization, and decrease the toxicity of CPA during the sperm cryopreservation process (Tian et al., 2015; Ahn et al., 2018). The current study did not reveal any effects of straw size on the post-thaw sperm motility when sperm were preserved with extender-1 or −2. Velasco-Santamaría et al. (2006) have also reported that the straw size has no effect on cryopreservation. In the present study, the post-thaw motility of 1-year cryopreserved sperm showed a significant similarity with the immediate cryopreserved (3-h) sperm and those of fresh sperm motility. Tanaka et al. (2002) reported that different storage periods showed a significantly similar post-thaw motility of Japanese eel. Similar phenomena were also reported from the cryopreserved turbot sperm (Suquet et al., 1998).

Cryopreservation with MeOH resulted in a decreased post-thaw sperm motility compared to that with Me_2_SO, GLY, EG, or PG. It also produced the lowest post-thaw motility for sperm of other flounder species such as *Paralichthys olivaceus* (Zhang et al., 2003), *Paralichthys dentatus* (Brown et al., 2012), and *V. variegatus* (Tian et al., 2008). Although it is associated with a low post-thaw motility of sperm of marine flounders, it has been reported as an effective CPA for cryopreserving sperm of freshwater fish (Lahnsteiner et al., 1997). Cryopreservation with Ringer's solution (extender-3) resulted in a lower post-thaw motility of sperm than cryopreservation with other types of the extender.

The present study revealed that extender-3 was not suitable for the cryopreservation of marbled flounder sperm. However, it has been previously reported that extender-3 is suitable for cryopreserving sperm of *Rasbora tawarensis* (Muchlisin et al., 2020) and yellow catfish, *Pelteobagrus fulvidraco* (Pan et al., 2008). The present findings suggest that the absence of sugar in extender-3 might be responsible for the reduced motility of post-thaw sperm, although a positive impact of sugar-based extenders (extender-1 and extender-2) on the sperm cryopreservation has been described in the previous section because such extender can additionally act as a non-penetrating CPA (Rusco et al., 2019). Similarly, extender-4 was not appropriate for the cryopreservation of marbled flounder sperm, although it resulted in improved motility than extender-3. This improvement might be due to a slight addition of sugar in the composition of extender-4. However, it has been previously reported that extender-4 is appropriate for the cryopreservation of sperm of filefish, *Thamnaconus modestus* (Le et al., 2008). These differences shed some light on the variation in the effectiveness of CPAs for sperm cryopreservation. Species-specific effectiveness of extender might also be responsible for such kinds of variation. Species-specific effects of extenders on cryopreservation have been previously reported for fish sperm (Le et al., 2011).

Plasma membrane integrity is the most investigated physiological parameter of cryopreserved fish sperm (Figueroa et al., 2016). The concept of sperm viability is associated with the intactness of the plasma membrane (Hernández-Avilés et al., 2019). In this study, 7-day cryopreserved sperm showed a similar morphology as fresh sperm under a fluorescent microscope. Six combinations of CPAs and extenders were used to assess their effects on PMI based on the post-thaw sperm motility. Among various combinations, extender-1 + 15% Me_2_SO and extender-1 + 12% GLY did not show any significant difference in PMI compared to PMI of fresh sperm (*p* > 0.05). However, slight differences in the integrity of the plasma membrane were observed between sperm cryopreserved with extender-2 + 15% Me_2_SO and those cryopreserved with extender-2 + 12% GLY. Notably, PMI values of summer flounder and Brazilian flounder showed significant differences (*p* < 0.05) between fresh and cryopreserved sperm (Lanes et al., 2008; Brown et al., 2012). In the present study, PMI of 1-year cryopreserved sperm using extender-1 combined with 15% Me_2_SO or 12% GLY showed a significant value similar to PMI values of 7-day cryopreserved sperm and those of fresh sperm. PMI values of rainbow trout cryopreserved sperm did not show significant differences when sperm were preserved in different storage periods (Pérez-Cerezales et al., 2010), which showed consistent results with the present findings. The present study suggests that a combination of 15% Me_2_SO or 12% GLY with extender-1 does not affect the integrity of the plasma membrane of the long-term cryopreserved sperm.

Six combinations of CPAs and extenders were also used to assess their effects on MMP as an index of the post-thaw sperm quality. There were no significant differences in MMP (*p* > 0.05) between fresh sperm and sperm cryopreserved (7 days) with extender-1 + 15% Me_2_SO or extender-1 + 12% GLY. However, 15% Me_2_SO + extender-2, 12% GLY + extender-2, 15% EG + extender-1, and 15% PG + extender-1 resulted in significant (*p* < 0.05) differences in MMP values compared to MMP values of fresh sperm. Therefore, cryopreservation may not affect the mitochondrial membrane of cryopreserved sperm. The MMP of cryopreserved sperm derived from Atlantic salmon (*Salmo salar*) was significantly (*p* < 0.05) lower than that of fresh sperm (Figueroa et al., 2019). Similar to MMP, the post-thaw sperm motility of Atlantic salmon was also significantly lower than that of fresh sperm. In the present study, MMP of 1-year cryopreserved sperm using extender-1 combined with 15% Me_2_SO or 12% GLY showed a significant value similar to MMP values of 7-day cryopreserved sperm and those of fresh sperm. Kim et al. (2016) reported the effects of long-term (3-year) cryopreservation on MMP of seven-band grouper. However, the present study demonstrated that post-thaw motility of cryopreserved sperm was similar to that of fresh sperm, which could explain the absence of difference in MMP between fresh and sperm cryopreserved (7 days and 1 year) using extender-1 + 15% Me_2_SO or extender-1 + 12% GLY.

In the present study, motility, PMI, and MMP were used as three indicators of sperm vitality. Results showed that 15% Me_2_SO or 12% GLY along with extender-1 was appropriate for the cryopreservation of marbled flounder sperm. DNA integrity is another important indicator of successful cryopreservation (Balamurugan et al., 2019; Kim et al., 2020a). The freeze–thaw process may damage the DNA integrity of cryopreserved sperm (Steele et al., 2000; Balamurugan et al., 2019). Thus, a comet assay was performed to assess the DNA integrity of 7-day and 1-year cryopreserved sperm in the present study. However, a previous study has reported that the sperm nucleus is stable and undamaged during a freeze–thaw process (Gwo et al., 2003). Based on PMI and MMP results, comet assay was performed using sperm cryopreserved (7 days and 1 year) with extender-1 in combination with 15% Me_2_SO or 12% GLY. Results of the comet assay showed that cryopreservation did not damage the integrity of sperm DNA. There was no fragmentation of DNA from the nucleus of 7-day and 1-year cryopreserved sperm. Similarly, it has been reported that gray mullet, Atlantic croaker, and rainbow trout, seven-band grouper sperm are not damaged by the freeze–thaw process after the immediate or long-term cryopreservation (Labbe et al., 2001; Balamurugan et al., 2019; Kim et al., 2020a). However, conflicting findings have been reported for the sperm of sea bream and sea bass, showing large quantities of fragmented DNAs in cryopreserved sperm (Zilli et al., 2003; Cabrita et al., 2005). Natural variations between species are responsible for the specific tolerance to cryo-damage, particular chromatin structure, and variation in extender applicability for sperm cryopreservation (Pérez-Cerezales et al., 2009).

Indicators of post-thaw sperm vitality such as motility, PMI, MMP, and DNA integrity of sperm cryopreserved (7 days and 1 year) with extender-1 along with 15% Me_2_SO or 12% GLY did not show any significant difference compared to those of fresh sperm. Based on the findings of these indicators, we recommend the use of lower concentrations of CPAs (12% GLY and 15% Me_2_SO) with extender-1 over the use of higher concentrations of CPAs with extender-2 (20% EG or PG) reported previously (Song et al., 2016).

## Conclusion

The present study suggests that a sucrose-based extender in combination with 15% Me_2_SO or 12% GLY is optimal for the cryopreservation of marbled flounder sperm. This study analyzed several parameters associated with vitality indicators, including post-thaw sperm motility, PMI, MMP, and DNA integrity, to demonstrate the effectiveness of each combination of CPA and extender. This study also reported that post-thaw vitality of 1-year cryopreserved sperm using sucrose-based extender in combination with 15% Me_2_SO or 12% GLY is significant similar to fresh sperm. The suggested cryopreservation protocol may be helpful for hatchery production as improvement in *P. yokohamae* sperm cryopreservation techniques is required to create a germplasm bank.

## Data Availability Statement

The original contributions presented in the study are included in the article/Supplementary Material, further inquiries can be directed to the corresponding author.

## Ethics Statement

Experimental protocols used were approved by Chonnam National University Animal Care and Use Committee (Approval No.: CNU IACUC-YS-2019-10; Approval date: December 10th, 2019). All fish experiments were performed according to Guidelines for the Care and Use of Laboratory Animals of the National Institutes of Health.

## Author Contributions

KK and SH conceptualized the study, wrote the original paper, and involved in the review and editing of the manuscript. KK, SH, SK, and YC performed the methodology and investigation. SH performed data curation, visualization, formal analysis, and software analysis. KK did supervision, validation, and fund acquisition. All authors have read and agreed to the published version of the manuscript.

## Conflict of Interest

The authors declare that the research was conducted in the absence of any commercial or financial relationships that could be construed as a potential conflict of interest.
