# Supplementary material for: Vital Analysis of Cryopreserved Sperm of Marbled Flounder, Pseudopleuronectes yokohamae
Source: Front Physiol. 2021 Jun 28;12:696737. doi: 10.3389/fphys.2021.696737 (PMC8273914; doi:10.3389/fphys.2021.696737)
Supplement: Supplementary file 1 [file Table_1.pdf]

**Supplementary Table 1.** Post-thaw sperm motility of sperm cryopreserved with different extenders in combination with different CPAs at various concentrations (*n* = 3).

| Cryoprotectant[B] | Concentration (%) | Motility (%) (mean ± *SD*) | | | |
|---|---|---|---|---|---|
| | | **Extender-1[A]** | **Extender-2[A]** | **Extender-3[A]** | **Extender-4[A]** |
| Fresh sperm | - | 93.0 ± 1.7[a] | 91.3 ± 1.2[a] | 82.7 ± 3.1[a] | 82.0 ± 2.0[a] |
| $Me_2SO$ | 5 | 55.6 ± 4.0[f] | 56.0 ± 5.2[e] | 29.0 ± 4.6[g] | 44.5 ± 2.3[d] |
| | 10 | 68.3 ± 3.5[d] | 69.0 ± 3.0[d] | 42.8 ± 2.3[e] | 61.0 ± 3.6[b] |
| | 12 | 76.5± 5.1[c] | 74.0 ± 5.6[c] | 67.4 ± 5.2[b] | 56.9 ± 4.5[bc] |
| | 15 | 86.0 ± 5.3[ab] | 85.7± 7.0[ab] | 41.4 ± 4.2[e] | 54.7 ± 4.6[c] |
| GLY | 5 | 63.3 ± 4.7[e] | 42.7 ± 4.7[fg] | 21.8 ± 3.7[h] | 18.3 ± 1.5[g] |
| | 10 | 71.3 ± 7.2[cd] | 70.0 ± 2.0[cd] | 29.3 ± 4.2[g] | 20.8 ±1.1f[g] |
| | 12 | 83.7 ± 6.6[b] | 83.3 ± 4.7[b] | 54.5 ± 3.1[d] | 25.1 ± 3.9[f] |
| | 15 | 58 ± 2.0[f] | 61.0 ± 2.6[e] | 37.0 ± 2.6[f] | 35.0 ± 2.6[e] |
| EG | 5 | 17.7 ± 2.5[h] | 20.0 ± 2.0[i] | 16. 7 ± 4.1[i] | 33.3 ± 2.3[e] |
| | 10 | 61.3 ± 5.0[ef] | 54.8 ± 3.0[e] | 22.0 ± 3.5[h] | 57.7 ± 2.5[bc] |
| | 12 | 66.1 ± 7.0[de] | 38.1 ± 2.0[g] | 12.3 ± 3.7[i] | 36.1 ± 2.0[e] |
| | 15 | 77.7 ± 5.6[bc] | 35.6 ± 2.1[gh] | 7.3 ± 2.3[j] | 18.2 ± 2.3[g] |
| PG | 5 | 31.6 ± 3.5[g] | 8.0 ± 2.0[j] | 36.0 ± 1.7[f] | 43.0 ± 2.6[d] |
| | 10 | 60 ± 5.2[ef] | 31.3 ± 3.1[h] | 41.3 ± 3.1[e] | 38.3 ± 3.5[de] |
| | 12 | 69 ± 4.1[cd] | 60.9 ± 3.1[e] | 58.4 ± 3.5[cd] | 24.6 ± 3.1[f] |
| | 15 | 77 ± 3.0[bc] | 70.7 ± 2.3[cd] | 62.8 ± 4.5[bc] | 10.5 ± 2.3[h] |
| MeOH | 5 | 65.8 ± 4.0[de] | 44.3 ± 4.0[f] | 45.9 ± 2.9[e] | 24.6 ± 2.5[f] |
| | 10 | 38.1 ± 4.4[g] | 38.9 ± 3.1[g] | 36.8 ± 3.8[f] | 33.0 ± 3.0[e] |
| | 12 | 9.3 ± 4.1[h] | 28.8 ± 1.3[h] | 12.4 ± 1.5[i] | 19.3 ± 3.8[g] |
| | 15 | 0.0 ± 0.0 | 5.7 ± 2.5[j] | 0.0 ± 0.0 | 0.0 ± 0.0 |

**Note:** [A]Values are expressed as mean ± SD (n = 3). Different letters (a, b, c, d, and e) indicate statistically significant differences within each extender ($p < 0.05$).

[B] $Me_2SO$, dimethyl sulfoxide; GLY, glycerol; EG, ethylene glycol; PG, propylene glycol; MeOH, methanol.
